# Fluid Dynamics Appearing during Simulated Microgravity Using Random Positioning Machines

**DOI:** 10.1371/journal.pone.0170826

**Published:** 2017-01-30

**Authors:** Simon L. Wuest, Philip Stern, Ernesto Casartelli, Marcel Egli

**Affiliations:** 1 Lucerne University of Applied Sciences and Arts, School of Engineering and Architecture, CC Aerospace Biomedical Science and Technology, Space Biology Group, Hergiswil, Switzerland; 2 Lucerne University of Applied Sciences and Arts, School of Engineering and Architecture, CC Fluid Mechanics and Hydraulic Machines, Horw, Switzerland; Coastal Carolina University, UNITED STATES

## Abstract

Random Positioning Machines (RPMs) are widely used as tools to simulate microgravity on ground. They consist of two gimbal mounted frames, which constantly rotate biological samples around two perpendicular axes and thus distribute the Earth’s gravity vector in all directions over time. In recent years, the RPM is increasingly becoming appreciated as a laboratory instrument also in non-space-related research. For instance, it can be applied for the formation of scaffold-free spheroid cell clusters. The kinematic rotation of the RPM, however, does not only distribute the gravity vector in such a way that it averages to zero, but it also introduces local forces to the cell culture. These forces can be described by rigid body analysis. Although RPMs are commonly used in laboratories, the fluid motion in the cell culture flasks on the RPM and the possible effects of such on cells have not been examined until today; thus, such aspects have been widely neglected. In this study, we used a numerical approach to describe the fluid dynamic characteristic occurring inside a cell culture flask turning on an operating RPM. The simulations showed that the fluid motion within the cell culture flask never reached a steady state or neared a steady state condition. The fluid velocity depends on the rotational velocity of the RPM and is in the order of a few centimeters per second. The highest shear stresses are found along the flask walls; depending of the rotational velocity, they can reach up to a few 100 mPa. The shear stresses in the “bulk volume,” however, are always smaller, and their magnitude is in the order of 10 mPa. In conclusion, RPMs are highly appreciated as reliable tools in microgravity research. They have even started to become useful instruments in new research fields of mechanobiology. Depending on the experiment, the fluid dynamic on the RPM cannot be neglected and needs to be taken into consideration. The results presented in this study elucidate the fluid motion and provide insight into the convection and shear stresses that occur inside a cell culture flask during RPM experiments.

## Introduction

The behavior and development of biological cells are largely governed by their complex interaction with external stimuli. Besides a variety of signals, including electrical and chemical signals, cells also respond to mechanical stimulations. Cells react to a variety of mechanical stresses, such as tensile, compressive and shear stresses [1–5]. The influence of mechanical stimulation on cells is of such great importance and complexity that a new research field called mechanomics evolved [6, 7]. In order for cells to respond to mechanical stimuli, they need to adhere through focal adhesion (FA) junctions [8–10], for example. Most cells communicate with the extracellular matrix by bridging FA with intracellular connectors such as fibronectin and other related bridge molecules. Research on cell adherence has revealed many findings on how cells can sense their environment and transduce mechanical forces into intracellular signals [2, 11].

As a fundamental and omnipresent force, gravity greatly influences the development of multicellular organisms, such as humans. At the onset of human space flight, it became clear that reduced gravity have profound effects on human health and physiology [12]. For instance, short and long-term space flights cause severe muscle wasting (atrophy) [12–15]. Additionally, certain shock-absorbing tissues, such as the intervertebral discs (IVD) of the spine, react to microgravity (or weightlessness) with increased water uptake and increased swelling. This is thought to be one major reason for the back pain frequently experienced by astronauts during space travel; it is also associated with the increased risk of disc herniation after prolonged space flights [16, 17]. The degenerative effect of microgravity on IVDs has been further demonstrated in space-flown experiments [18–20].

In order to study the influence of gravity on biological systems (in the field of gravitational biology), various technical platforms are employed in order to expose samples to either hypergravity or lowgravity. While hypergravity experiments can be conducted quite easily through centrifuges, lowgravity experiments tend to be more problematic. Depending on the desired lowgravity exposure duration, various research platforms, such as drop towers, parabolic flights and space flights, can be used. Due to the extensive preparation efforts of space-flown experiments, ground-based microgravity simulation models are frequently used as a test method or as an alternative to space flights. As such, the clinostat and the Random Positioning Machine (RPM) are among the most successful machines and are widely used around the world [21].

RPMs basically consist of two gimbal mounted frames that constantly rotate biological samples around two perpendicular axes. Their working principle is based on gravity vector averaging to zero [22]. Through dedicated algorithms, the gravity vector is distributed in all directions over time. Therefore, observed from the samples’ point of view, the gravity vector’s trajectory averaged over time converges to zero. It is assumed that the Earth’s gravity vector needs to point in a specific direction for a minimum amount of time in order to allow biological samples to adapt to gravity. If the samples are rotated constantly and fast enough, no adaptation will be possible anymore; thus, they will experience a “microgravity-like condition” [23]. Experiments on the RPM have shown comparable results to space-flown experiments in several studies (reviewed in [24]). Despite the fact that the RPM was originally developed for gravitational research, it has become an interesting tool for other mechanobiological research and applications as well. For example, the RPM has been used to simulate a mechanical disuse condition and provoke decreased bone formation in preosteoblast cells [25]. Further spheroid cell clusters could be formed on the RPM with several cells, making the machine a promising approach to generating scaffold-free three-dimensional cell constructs (reviewed in [26]). These spheroids are highly interesting for cancer research (reviewed in [27]) or for tissue engineering. For example, the RPM has recently been used to create scaffold-free human articular chondrocyte cell clusters [28].

The kinematic rotation of the RPM does not only distribute the Earth’s gravity vector over time, but it also introduces local forces to the cell culture containment, which can be described by rigid body analysis [24, 29]. Because these forces are not fully controllable, scientists are advised to place the samples close to the center of rotation in order to minimize them [24]. However, to date, the fluid motion within a culture chamber and its possible effects on the cells have been the subject of very few publications and have only been discussed rudimentarily [30]. When filming macroscoping particles inside a water-filled culture chamber that is mounted on an operating RPM, rapid fluid motion can be observed (unpublished observations). The fluid appeared to frequently invert the flow direction within the chamber. In this study, we used a numerical approach to describe the complex fluid dynamic characteristic inside such culture chambers on an RPM in order to elucidate the actual stresses working on the cells. Such analysis is crucial when interpreting the biological results from the RPM. Thus, it should be taken into consideration for every biological experiment involving the use of an RPM as a microgravity simulation tool.

For explanatory reasons and for the sake of completeness, the fluid dynamics appearing on the two simpler and related machines, the centrifuge and the clinostat, are discussed as well. Both rotate their samples around or on a single axis, respectively. Centrifuges are typically used for hypergravity experiments, rotating around a vertical axis with the samples being a certain radius from the rotational axis. Clinostats represent an alternative model for microgravity simulation. They rotate about a horizontal axis, and the samples are fixed on the rotational axis in order to avoid centrifugal forces.

## Numerical Methods

### Geometry and Setup

For clinostat and RPM experiments, the biological samples are kept in either custom-made containers or in commercially available flasks, which are entirely filled with cultivation medium (bubble-free). Despite the chemical additives, the cultivation medium is very much aqueous. Therefore, the cultivation medium was represented by water at 37°C, implying a density of 993.336 kg/m^3^ and a dynamic viscosity of 6.91519·10^-4^ Pa·s. The cell culture flask was modeled as a rectangular box (55.56 x 45 x 25 mm), which roughly approximated the shape of a commercially available T25 flask (culture surface of 25 cm^2^). In order to separate the shear stresses along the wall from the shear stresses within the bulk volume during post processing of the results, two domains were defined. The outer domain represents the flask geometry. The inner domain, being 4 mm smaller than the flask (51.56 x 41 x 21 mm), is referred to as the “bulk volume.” The geometry was designed with the CAD software “NX8” (Siemens).

All simulations were performed with “ANSYS CFX 16.0” (ANSYS, Inc.) using a second order upwind scheme and the shear stress transport (SST) turbulence model with transition modeling, as explained in more detail below. The mesh consisted of 4.014 million hexa elements and was generated with the software “Pointwise V17.3R3” (Pointwise, Inc.). A first wall distance of 7.5 μm assured a y^+^ smaller than 1 for all cases. Using a low-Reynolds mesh resolution (i.e. no adoption of wall functions) with y^+^ being smaller than 1 and a growing ratio for the mesh of 1.06, it is ensured that boundary layer and core are properly resolved. This is also a requirement for the used SST model with transition. Accordingly the shear stresses at the walls are computed from the boundary and the local flow conditions, i.e. not using universal wall functions based on flat plate equilibrium conditions. Using this approach and the above mentioned growth rate, leads to a fine mesh up to the center of the flask. A mesh dependency study was therefore not considered necessary.

The RPM’s rotation about two perpendicular axes was accomplished with the approach of mesh movement, where the corresponding displacement was given by expressions representing the rotation matrix in three dimensions. So the orientation of the mesh was calculated for every time step and the high quality of the hexa mesh was conserved. In order to simplify the setup, the rotational velocities of the two axis were assumed to be constant and identical. In these transient simulations, a one-time step covers one degree of rotation. This means that in the case of a rotating velocity of 40 deg/s, the time step is 25 ms; for 60 deg/s, it is 16.66 ms, and for 90 deg/sec, it is 11.11 ms. The Earth’s gravity vector was defined as pointing in the negative z-direction (-9.81 m/s^2^). This method was chosen in regard to the fact that vector rotations in ANSYS CFX are only realizable around one axis and not two, which is inevitable for simulating an RPM. The container walls were defined as no-slip boundary conditions. The initial internal pressure was set to 1 bar.

### Governing Equations

The simulations were performed solving the Navier Stokes equation for incompressible flow using a pressure based formulation. It consists of continuity and momentum equations, respectively. (Refer to Table 1 for the declaration of the variables in the equations.)

**Table 1:** Declaration of the variables used in the equations.

Symbol	Description
*E*	Transition sources
*g*	Earth gravity (9.81 m/s^2^)
*P*	Transition sources
*p*	Pressure
$\vec{p}$	Position vector
*r*	Radius
${\tilde{Re}}_{\theta t}$	Critical Reynolds number
*R*_*x*_, *R*_*y*_, *R*_*z*_	Rotation around the x- y- or z-axis, respectively
*S*_*M*_	Momentum source
*t*	Time
*U*	Velocity vector
*u*	Fluctuating velocity component in turbulent flow
*α*	Inclination towards the rotational axis
*γ*	Intermittency
*μ*	Molecular (dynamic) viscosity
*μ*_*t*_	Turbulent viscosity
*ρ*	Density
*σ*	Constant
*τ*	Molecular stress
*ω*	Angular velocity
*δ*_*ij*_	Kronecker delta
$\rho \bar{u_{i}u_{j}}$	Reynolds stresses

$$\frac{\partial \rho }{\partial t}+\frac{\partial }{\partial x_{j}}(\rho U_{j})=0$$ (1)

$$\frac{\partial \rho U_{i}}{\partial t}+\frac{\partial }{\partial x_{j}}(\rho U_{i}U_{j})=\frac{\partial p}{\partial x_{i}}+\frac{\partial }{\partial x_{j}}(\tau_{ij}-\rho \bar{u_{i}u_{j}})+S_{M}$$ (2)

Due to the uncertainty of the flow’s characteristic, a turbulence model capable of transition was used [31, 32]. This approach ensures maximum flexibility and accuracy: depending on the local flow conditions the model uses laminar or turbulent flow modeling, thus ensuring a correct treatment of the dissipation in the flask. The Reynolds number for the simulations 40 deg/s, 60 deg/s and 90 deg/s were 1434, 2172 and 3267 respectively. They were calculated by Ansys CFX using the cube root of the volume to calculate the length scale. The shear stress transport (SST) turbulence model is a two-equation model, where the Re-stresses are computed using the Boussinesq assumption [31, 32]. In addition the Gamma-Theta transition model was used, which is based on two additional equations: the transport equation for the intermittency, and the transport equation for the transition momentum thickness Reynolds number, respectively.

$$\frac{\partial (\rho \gamma )}{\partial t}+\frac{\partial (\rho U_{j}\gamma )}{\partial x_{j}}=P_{\gamma 1}-E_{\gamma 1}+P_{\gamma 2}-E_{\gamma 2}+\frac{\partial }{\partial x_{j}}\left[(\mu +\frac{\mu_{t}}{\sigma_{\gamma }})\frac{\partial \gamma }{\partial x_{j}}\right]$$ (3)

$$\frac{\partial (\rho {\tilde{Re}}_{\theta t})}{\partial t}+\frac{\partial (\rho U_{j}{\tilde{Re}}_{\theta t})}{\partial x_{j}}=P_{\theta t}+\frac{\partial }{\partial x_{j}}\left[(\sigma_{\theta t}(\mu +\mu_{t})\frac{\partial {\tilde{Re}}_{\theta t}}{\partial x_{j}})\right]$$ (4)

The interested reader is referred to the publications of Menter and Langtry [31, 32] for more details.

#### Centrifuge

**Fig 1:**
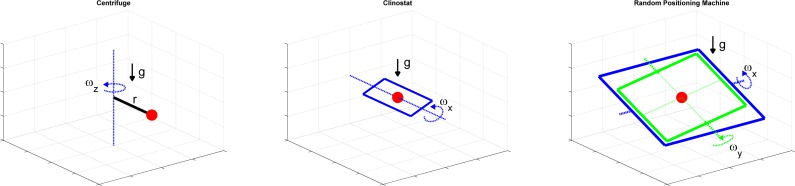
Schematic illustration of the working principles of the centrifuge (left), clinostat (middle) and Random Positioning Machine (RPM, right). Centrifuges are used for hypergravity experiments. Thereby, the sample (red dot) is rotated around a vertical axis within a certain radius from the axis. Clinostats and RPMs are used for simulated microgravity experiments. Whereas the clinostat rotates the samples around one horizontal axis, the RPM rotates the samples around two perpendicular axes.

On the centrifuge, the samples are rotated about a vertical axis with a certain radius *r* in order to expose them to increased acceleration (Fig 1). The governing equation for the centrifuge’s rotation is therefore:

$$\vec{p}(t)=R_{z}∙{\vec{p}}_{t0}$$ (5)

$$\vec{p}(t)=\left[\begin{array}{ccc} cos(\omega ∙t) & sin(\omega ∙t) & 0\\ -sin(\omega ∙t) & cos(\omega ∙t) & 0\\ 0 & 0 & 1 \end{array}\right]∙\left(\begin{array}{c} x_{t0}\\ y_{t0}\\ z_{t0} \end{array}\right)$$ (6)

where $\vec{p}(t)$ represents the position of any arbitrary point in space at the time *t*, which was at the point ${\vec{p}}_{t0}$ at *t* = 0 and is transformed under the angular velocity *ω*.

On a centrifuge, the samples are normally mounted on a swinging gondola. This ensures that the flask will always orient itself normally to the resulting acceleration vector. Therefore, the culture flask is inclined toward the rotational axis by the angle *α*:

$$\alpha =\tan^{-1}\left(\frac{\omega^{2}∙r}{g}\right)$$ (7)

where *r* is the radial distance to the rotation axis and *g* is the Earth’s gravity (*g* = 9.81 m/s^2^).

#### Clinostat

The clinostat rotates the samples around a horizontal axis. In order to avoid centrifugal forces, the samples are normally placed exactly on the rotational axis (Fig 1). Using the same convention as above, the governing equation is:

$$\vec{p}(t)=R_{x}∙{\vec{p}}_{t0}$$ (8)

$${\vec{p}(t)}_{}=\left[\begin{array}{ccc} 1 & 0 & 0\\ 0 & cos(\omega ∙t) & -sin(\omega ∙t)\\ 0 & sin(\omega ∙t) & cos(\omega ∙t) \end{array}\right]∙\left(\begin{array}{c} x_{t0}\\ y_{t0}\\ z_{t0} \end{array}\right)$$ (9)

#### Random positioning machine

The RPM rotates the samples around two perpendicular axes (Fig 1). Depending on the RPM’s developer, the algorithms responsible for distributing the gravity vector are different [24]. One previously implemented algorithm lets the frames rotate at a constant speed and inverts the rotating direction at random points in time [29]. As mentioned above, for this study, only the simplified case, where the two frames’ rotation is constant and equal, is used. Similar to above, the governing equation is:

$$\vec{p}(t)=R_{x}∙R_{y}∙{\vec{p}}_{t0}$$ (10)

$${\vec{p}(t)}_{}=\left[\begin{array}{ccc} 1 & 0 & 0\\ 0 & cos(\omega ∙t) & -sin(\omega ∙t)\\ 0 & sin(\omega ∙t) & cos(\omega ∙t) \end{array}\right]∙\left[\begin{array}{ccc} cos(\omega ∙t) & 0 & sin(\omega ∙t)\\ 0 & 1 & 0\\ -sin(\omega ∙t) & 0 & cos(\omega ∙t) \end{array}\right]∙\left(\begin{array}{c} x_{t0}\\ y_{t0}\\ z_{t0} \end{array}\right)$$ (11)

### Post Processing

The following quantities were extracted from the simulations: (1) the average and maximum values of the relative fluid velocity within the flask; (2) the maximum shear stress appearing on the two largest surfaces, on which normally adherent cells are cultured—therefore, we refer to them as the “cultivation surface”; (3) the ratio of the area of the cultivation surface, which is exposed to a shear stress exceeding defined thresholds; (4) the average and maximum shear stress in the “bulk volume”; and (5) the mixing (convection) induced by the fluid motion.

The relative fluid velocity within the flask was calculated by subtracting the mesh velocity, representing the rigid body velocity due to the rotation around the two axes, from the absolute fluid velocity in the global coordinate system. For averaging, the volume average was used.

In order to analyze the mixing quality of the RPM, the flask domain was split in two along the xz-plane at y = 0. We refer to these two compartments as the “y-positive compartment” (for y ≥ 0) and the “y-negative compartment” (for y < 0). A virtual variable was implemented at the concentration of 10 units/m^3^ in the “y-positive compartment” only. Thus, the concentration of the variable in the “y-negative compartment” was 0 units/m^3^. Subsequently, the variable was left to spread by convection over three periods. To monitor the concentration of the variable over time, 1000 monitor points were equally distributed in the flask (10 points along each axis). After the simulations, for every time step, the concentration average of the two compartments was computed.

### Limitations

**Fig 2:**
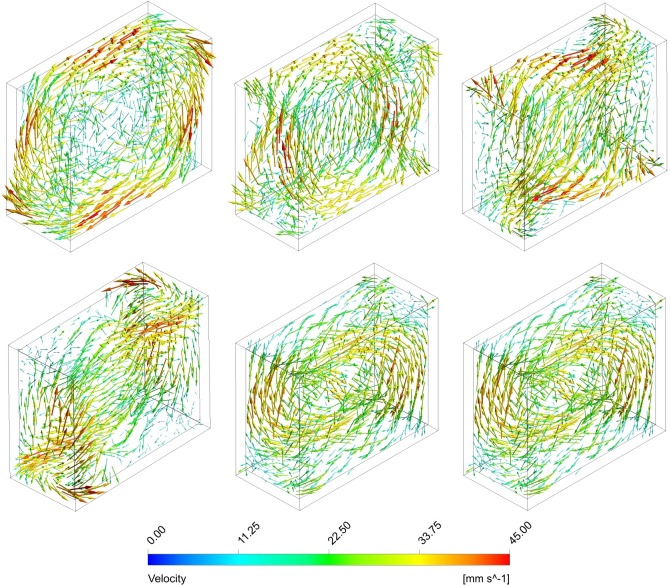
Velocity profile during one period on the RPM. Both frames rotate with constant and equal velocity; the flask is placed at the center of rotation. The rotational velocity is 60 deg/s. The time interval between the illustrations is 1 second (top left: 0 s; top middle: 1 s; top right: 2 s; bottom left: 3 s; bottom middle: 4 s; bottom right: 5 s).

**Fig 3:**
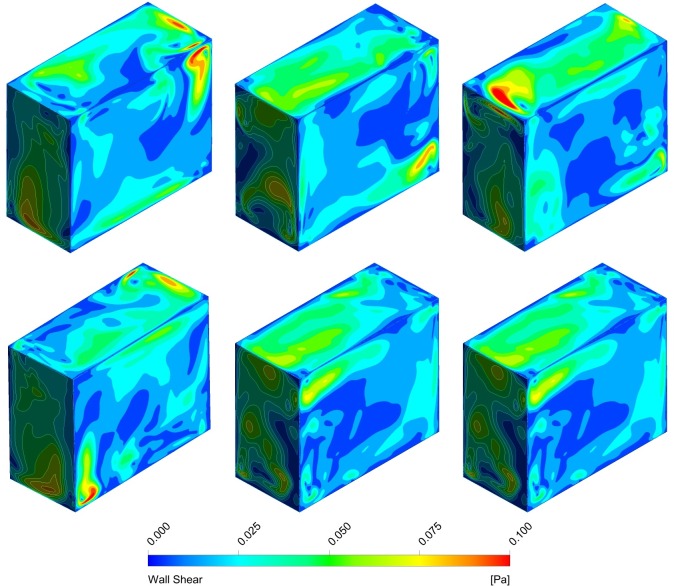
Shear stresses along the walls during one period on the RPM. Both frames rotate with constant and equal velocity; the flask is placed at the center of rotation. The rotational velocity is 60 deg/s. The time interval between the illustrations is 1 second (top left: 0 s; top middle: 1 s; top right: 2 s; bottom left: 3 s; bottom middle: 4 s; bottom right: 5 s).

For the centrifuge and the clinostat, the mechanics is straightforward and depends mainly on the rotational velocity and the radius from the rotational axis. The mechanics of the RPM is considerably more complex because it depends on the rotational velocities of both frames and the exact position of the sample in space. In order to reduce the solution space substantially, we restricted ourselves to the simplified case, where both frames rotate at a constant and equal speed and when the flask is placed at the center of rotation. In a screening study, the orientation of the flask leading to maximal shear stresses (worst case) was determined. All results presented below refer to the following orientation: At the initial position (*t*_*0*_), the edges are aligned with the axis of the global coordinate system such that the longest side of the flask is parallel to the x-axis and the shortest side is parallel to the y-axis (Figs 2 and 3). Furthermore, we do not discuss the influence of the flask’s geometry on the fluid dynamics in this study.

## Results

### Centrifuge

After centrifuges reach nominal speed, a steady-state condition is established. After an initial phase, the motion of the liquid within the culture flask also becomes steady and can be described with rigid body analysis. Thus, there is no relative velocity motion within the culture flask and therefore no shear stresses appear. In centrifuge experiments, the samples are normally placed within a certain radius to the rotational axis in order to increase the centrifugal acceleration (*a* in m/s^2^) according to *a* = *ω*^2^ ∙ *r*, where *ω* is the rotation velocity (in rad/s) and *r* is the radial distance from the center of rotation (in meters). In order to avoid spatial gradients of the radial forces within the culture flask, it is beneficial to minimize the size of the culture flask and maximize the radius of the centrifuge [33]. The simulations, which were conducted with radii of 0, 10, 20 and 30 cm, showed no detectable effects that could be explained by spatial force gradients. The small residual velocity detectable in the simulations was attributed to numerical imprecisions.

### Clinostat

In comparison to centrifuges, the samples in clinostat experiments are ideally placed exactly on the rotational axis in order to avoid centrifugal forces [34]. Additionally, the rotational axis is horizontal, meaning that the gravitational force from the samples’ point of view is no longer steady (as in centrifuges), but rotating. Nevertheless, the medium within the flask becomes steady after an initial phase and can be described by rigid body analysis [34]. The simulations did not show any effect of the rotating gravity vector (from the samples’ point of view) on the fluid motion.

### Random Positioning Machine

For RPM experiments, scientists are advised to place their samples close to the center of rotation in order to minimize centrifugal accelerations [24]. Because multiple culture flasks are often placed on the RPM, not all of them can be placed at the center of rotation. Also, flasks can be placed in any arbitrary orientation on the RPM. This makes the possible solution space of the numerical simulations very large. To reduce complexity, an analysis for the worst case with the highest shear stress was done. For simplification reasons, the culture flask was placed at the center of rotation in the simulations. Our analysis showed that the highest shear stresses appeared if the flask was oriented with its longest side parallel to the x-axis and its shortest side parallel to the y-axis at the initial position (Fig 2) when rotating around the x and y axes.

**Fig 4:**
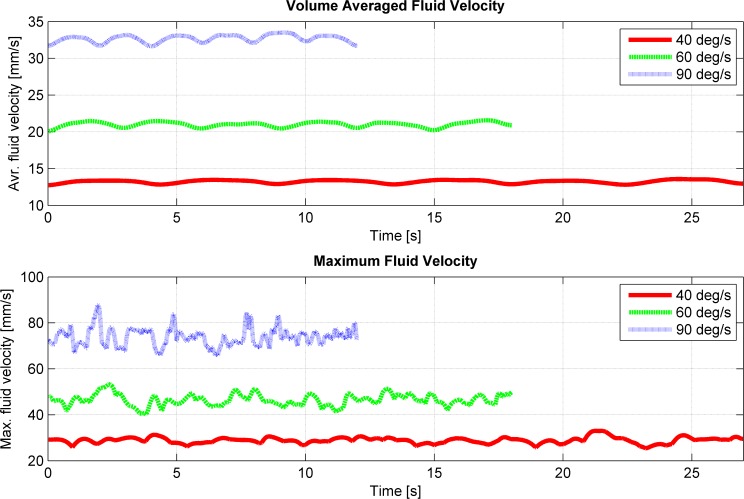
Velocity of the fluid in the flask during three periods on the RPM for three different rotational velocities (40, 60 and 90 deg/s). Both frames rotate with constant and equal velocity, and the flask is placed at the center of rotation. Top: Volume average of the velocity plotted over time. Bottom: Fastest velocity plotted over time.

**Fig 5:**
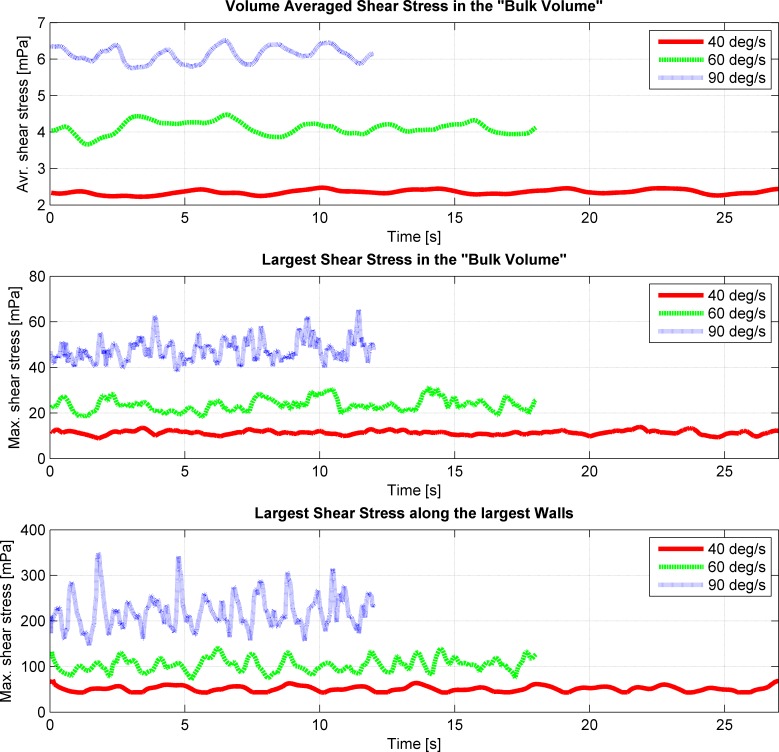
Shear stresses in the flask during three periods on the RPM for three different rotational velocities (40, 60 and 90 deg/s). Both frames rotate with constant and equal velocity, and the flask is placed at the center of rotation. Top: Volume average of the shear stresses in the “bulk volume” over time. The “bulk volume” is 4 mm smaller than the flask and thus has a 2 mm clearance from the flask wall. Middle: Maximum shear stresses in the “bulk volume” over time. Bottom: Maximum shear stresses along the “cultivation surface” (the two largest flask walls).

**Fig 6:**
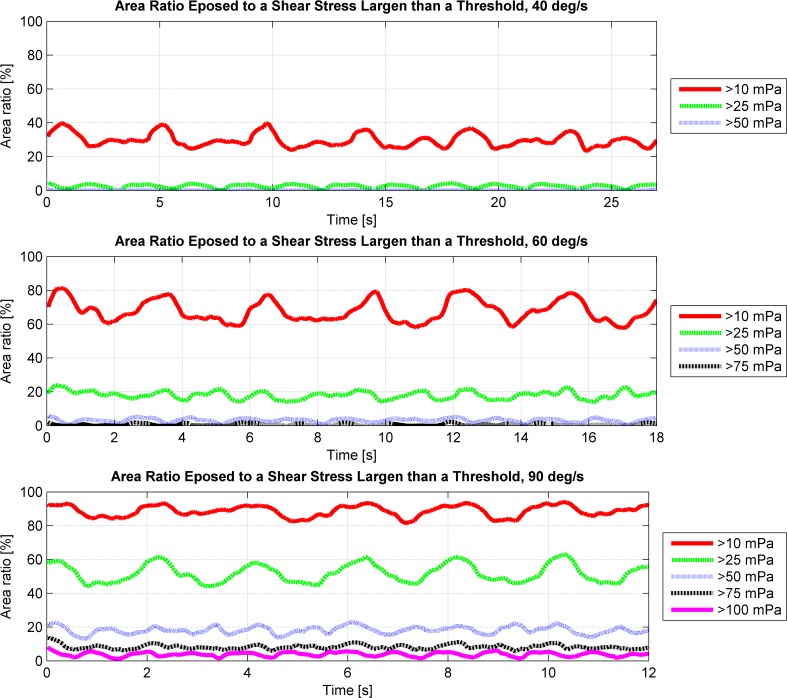
The ratio of the “cultivation surface” (the two largest flask walls) exposed to a shear stress larger than a certain threshold. The thresholds are chosen at 10, 25, 50, 75 and 100 mPa. The time represents three periods on the RPM for three rotational velocities (40, 60 and 90 deg/s). Both frames rotate with constant and equal velocity, and the flask is placed at the center of rotation.

The simulations showed that the fluid motion within the cell culture flask on the RPM never reached a steady state or neared a steady state condition (Figs 2 and 4), as was the case on the centrifuge and the clinostat. The circulating fluid periodically inverted its circulation direction. This observation can be explained by the following consideration: The rotating flask will transfer its momentum into the fluid, leading to a circulating fluid similar to the centrifuge or the clinostat. Since the RPM rotates around two axes, the rotation of the flask will soon not coincide with the rotation of the fluid motion anymore and will actually oppose the fluid circulation. Therefore, the fluid motion will eventually be inverted. By the time the fluid picks up velocity again, the flask will have moved farther and will soon oppose the fluidic circulation again. If both frames of the RPM rotate with a constant velocity (as done in our analysis), a periodically inverting circulating fluid motion can be observed in the flask. Because the momentum transfer happens mainly in the boundary layer along the flask wall, the highest shear stresses are also found along the flask wall (Figs 3 and 5). The point experiencing the highest shear stresses moves along the flask wall over time (Fig 3). Since many cell culture analyses are done on a population of cells, the effects experienced by individual cells are averaged out. Assuming that adherent cells are evenly distributed on the cultivation area, the ratio of cells experiencing a shear stress greater than a certain threshold can be computed (Fig 6). With increasing rotational velocity, the ratio of cells experiencing a certain minimal shear stress increases. For instance, less than 5% of the cells are exposed to a shear stress greater than 25 mPa at 40 deg/s. The percentage, however, increases to about 18% when rotating at 60 deg/s and to about 50% at 90 deg/s (Fig 6).

**Fig 7:**
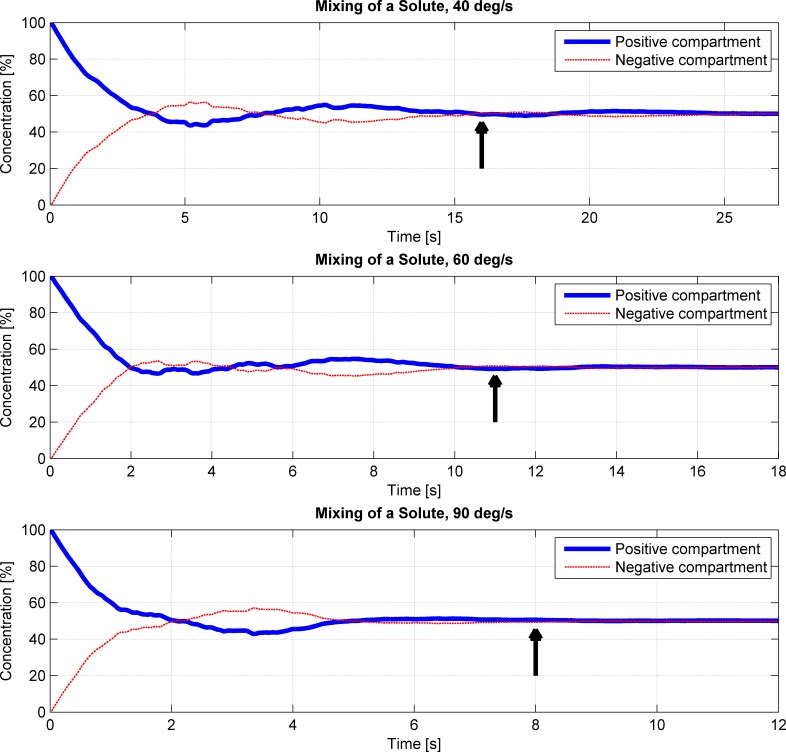
Convection on the RPM over three periods for three different rotational velocities (top: 40 deg/s; middle: 60 deg/s; bottom: 90 deg/s). Both frames rotate with constant and equal velocity, and the flask is placed at the center of rotation. The flask was divided into two compartments, denoted as the “y-positive compartment” (for y ≥ 0) and “y-negative compartment” (for y < 0). A virtual variable was placed in the “y-positive compartment” only. Subsequently, the variable was left to mix by convection, and the average concentration in the two compartments was monitored. The rapid fluid motion leads to thorough mixing within two to three periods (arrows).

The fluid motion in the cell culture flask does not only lead to shear stresses, but also to a very rapid mixing (convection) of the medium (Fig 7). The simulations showed that thorough mixing is achieved only within two periods. Depending on the rotational velocity, complete mixing is established within 8 seconds at 90 deg/s, or within 16 seconds at 40 deg/s (arrows in Fig 7).

## Discussion and Conclusion

Even though RPMs have been in use for several years, the fluid dynamic appearing in the culture flask has never been analyzed deeply; thus, it has been widely neglected. Besides distributing the Earth’s gravity vector, the motion of the RPM induces enhanced convection and increased shear stresses. In this study we elucidated the fluid velocity, shear stresses and convection for a specific and simplified case: (1) The flask has the approximate geometry of a standard T25 cell culture flask. (2) It is placed at the center of rotation. (3) The two frames of the RPM rotate with equal and constant velocity. Multiple RPMs have been developed with different controlling algorithms implemented. Some RPMs rotate the frames with random velocities, while other RPMs rotate with constant velocities, inverting periodically the rotational direction (reviewed in [24]). Therefore, some deviation from the results presented here should be expected on the various RPM systems. In general, larger shear stresses can be expected with increasing rotational velocities. The largest shear stresses in the “bulk volume” are in the order of a magnitude of 10 mPa. The highest shear stresses always appear along the flask’s wall. In the simulations, they were around 50 mPa, 100 mPa and 200 to 300 mPa for rotational velocities of 40 deg/s, 60 deg/s and 90 deg/s, respectively. Thus, the shear stress appearing on the RPM was in the range that provoked a cellular effect in previously published experiments (compare to Table 2). However, only a small portion of the cell population is exposed to the highest shear stresses along the flask walls. This means that for adherent cells less than 5% of the cell population is exposed to shear stresses greater than 50 mPa for rotational velocities up to 60 deg/s. Even rotational velocities up to 90 deg/s do not expose more than 5% of the cell population to shear stresses greater than 100 mPa.

**Table 2:** Effects of shear stresses on various cells.

Shear	Cell	Effect	Ref.
1.3…75.3 mPa	ENaC overexpressing Oocytes	Activated Epithelial Na^+^ Channels (ENaC)	[35]
20…400 mPa	Vascular endothelial cells	Modulated cytosolic-free calcium	[36]
20…17000 mPa	Endothelial cell	Activated K^+^ current	[37]
60…1500 mPa	Aortic endothelial cells	Modulated expression of Cu/Zn superoxide dismutase	[38]
100…1500 mPa	Aortic endothelial cells	Increases in pinocytotic rate	[39]
120…1500 mPa	Endothelial cell	Induction of ecNOS mRNA in a dose-dependent manner	[40]
150…1000 mPa	Embryonic stem cells	Increased the expression of vascular endothelial cell-specific markers at the protein level and the mRNA level	[41]
160, 410, 820 mPa and 1.64 Pa	Chondrocytes	Upregulated nitric oxide, membrane phosphatidylserine and nucleosomal degradation	[42]
200 mPa	Arterial vascular endothelial cells	Induction of endothelial stress fibers	[43]
350…11700 mPa	Endothelial cells	Stimulated mitogen-activated protein kinase	[44]
500…1000 mPa	Aortic endothelial cells	Changes in cell morphology	[45]
500…2500 Pa	Aortic smooth muscle cells	Reduced proliferation rate	[46]
600…2500 mPa	Endothelial cells	Induced nitric oxide production	[47]
600…2600 mPa	Endothelial cells	Reorganization of the cytoskeleton	[48]
800…1500 mPa	Vascular endothelial cell	Cell alignment in the direction of flow without initiating the cell cycle	[49]
1 Pa	Osteoblasts	Induced β-catenin signaling	[50]
1…2 Pa	Smooth muscle cell	Inhibited migration	[51]
1, 2 Pa	Osteoblastic cells	Produced higher magnitude and more abundant [Ca^2+^]_i_-oscillations than spontaneously	[52]
1, 3, 8.5 Pa	Endothelial cells	Shape change and cytoskeleton reorganization	[53]
1…8.5 Pa	Vascular endothelial cells	Orientation with the flow direction	[54]
1.2 Pa	Endothelial cells	Reorganization of the surface topography	[55, 56]
1.2 Pa	Vascular endothelial cells	Enhanced activation of transcription factors	[57]
1.5 Pa	Venous endothelial cells	Stimulated phosphorylation of Akt	[58]
1.52 Pa	Endothelial cells	Reorganization of the cytoskeleton	[59]
1.6 Pa	Chondrocytes	Down-regulation of the aggrecan gene expression	[60]
1.6 Pa	Chondrocytes	Stimulated glycosaminoglycan synthesis	[61]
1.64 Pa	Chondrocytes	Upregulated NO synthase gene expression and increased NO release; inhibited type II collagen and aggrecan mRNA levels	[62]
1.7…2.0 Pa	Osteoblast-like SaOS-2 cells	Increased TGF-β1 mRNA expression	[63]
2 Pa	Endothelial cells	Shape change and cytoskeletal remodeling	[64]
2 Pa	Osteoblast	Increased proliferation	[65]
3.5 Pa	Chondrocytes	Promoted chondrocyte proliferation	[66]

Nevertheless, RPMs and clinostats are much appreciated tools in microgravity-related research [21]. On the RPM, several cell types showed similar effects as under real microgravity in space [24]. Furthermore, the RPM is becoming a frequently used instrument in non-space-related research fields, as the RPM can be used, for instance, for three-dimensional cell culturing (spheroid cell cluster) [26, 27] and in tissue engineering. The results presented here suggest, however, that experiments on the RPM need to be designed carefully. Having a static condition as a control group, as done in most experiments, does not allow one to separate gravitational from fluid dynamic effects. The enhanced convection supplies the cells quickly with fresh medium and transports secreted (waste) products away. This can have a strong influence on cell growth and cell behavior in general. Especially on the RPM, the mixing is very efficient (Fig 7). In addition, shear stresses appearing within the cell culture flask can affect the cells as well. Since the maximum shear stresses always appear along the flask’s wall, adhered cells are generally exposed to higher shear stresses than suspended cells. Suspended cells, which stay in the “bulk volume,” are, at maximum, exposed to shear stresses in the order of a magnitude of 10 mPa. This does not hold if the cells temporarily come in close proximity to the flask’s walls, either through sedimentation or convection. Adherent cells, in contrast, are exposed to maximal shear stresses around 50 mPa, 100 mPa and 300 mPa for rotational velocities of 40 deg/s, 60 deg/s and 90 deg/s, respectively. Depending on the rotational velocity, about 20% or more of the cell population is exposed to shear stresses larger than 10 mPa for rotational velocities of 40 deg/s, 25 mPa for 60 deg/s and 50 mPa for 90 deg/s. For experiments that require one to distinguish between gravitational and fluid dynamic effect, we therefore propose to introduce an additional control condition with cells being exposed to fluid movements, such as on a swing or a rocker. Both machines cause enhanced convection but will not rotate the cell culture flask upside down. Therefore, the Earth’s gravity vector always points predominantly in one direction. However, swings and rockers cannot fully mimic the velocity profile seen on RPMs.

Several scientists have excluded fluid motion from gravitational effects by using controls placed on a shaker. In several studies, the test samples were compared to shaken control samples in clinostat [67–70] and RPM experiments [71, 72]. Introducing a shaken control beside the static control allows one to make a distinction between gravitational and fluid-mixing effects. Mouse embryonic stem cells showed increased proliferation on the clinostat and the shaker compared to a static control [73]. However, in the same study, these cells revealed a decreased differentiation ability, which could not be explained by simple mixing. Mouse myoblast showed increased cell proliferation and modest inhibition of differentiation on the clinostat, which could be partly explained by a stirred control group [74]. A study with an *Arabidopsis thaliana* cell line detected an increased number of cells in the G_1_ phase on clinorotated and shaken samples. At the same time, only in the clinostat group, a decreased nucleolus area staining could be observed, but not in the shaken and non-shaken control groups [75]. Similar several RPM experiments on mammalian cells have shown effects due to RPM exposure, which could not be explained by fluid dynamic medium mixing alone [76–78].

In conclusion, the RPM and the clinostat are well known as reliable tools in gravitational research. Furthermore, they have the potential to expand into new applications of the mechanobiological research field. However, depending on the experiment, the fluid dynamic on the RPM cannot be neglected and needs to be taken into consideration while designing the experiment and analyzing the results. Using appropriate control groups, this can give clues to possible undesired side effects. Due to the nature of the RPM, fluid motion within the cell culture flask is not avoidable. Nevertheless, the results presented here now provide scientists with an idea of the convection and shear stresses that have to be expected for such experiments.
